# Fast and reliable molecular methods to detect fungal pathogens in woody plants

**DOI:** 10.1007/s00253-020-10395-4

**Published:** 2020-01-31

**Authors:** Nicola Luchi, Renaud Ioos, Alberto Santini

**Affiliations:** 1 Institute for Sustainable Plant Protection, National Research Council (IPSP-CNR), Via Madonna del Piano, 10, I-50019 Sesto Fiorentino (Firenze), Italy; 2 ANSES Plant Health Laboratory, Unit of Mycology, Domaine de Pixérécourt, 54220 Malzéville, France

**Keywords:** Early detection, DNA-based techniques, Fungal pathogens, Invasive species, Plant biosecurity, Surveillance

## Abstract

Plant diseases caused by pathogenic microorganisms represent a serious threat to plant productivity, food security, and natural ecosystems. An effective framework for early warning and rapid response is a crucial element to mitigate or prevent the impacts of biological invasions of plant pathogens. For these reasons, detection tools play an important role in monitoring plant health, surveillance, and quantitative pathogen risk assessment, thus improving best practices to mitigate and prevent microbial threats. The need to reduce the time of diagnosis has prompted plant pathologists to move towards more sensitive and rapid methods such as molecular techniques. Considering prevention to be the best strategy to protect plants from diseases, this review focuses on fast and reliable molecular methods to detect the presence of woody plant pathogens at early stage of disease development before symptoms occur in the host. A harmonized pool of novel technical, methodological, and conceptual solutions is needed to prevent entry and establishment of new diseases in a country and mitigate the impact of both invasive and indigenous organisms to agricultural and forest ecosystem biodiversity and productivity.

## Introduction

Alien pathogens are exponentially increasing, challenging the sustainability of agriculture and forestry crops and natural ecosystems. The major pathway of non-native plant pathogen introduction is the international trade of plants, mainly ornamental (Liebhold et al. 2012; Santini et al. 2013). For these reasons, biosecurity protocols for plant protection have been developed to prevent the spread and assist in the eradication of invasive species (Bergeron et al. 2019; Wittenberg and Cock 2001). Previous experience from EU-funded projects identified critical points that should be tackled to improve the efficacy of the EU plant health regime. Despite the high costs of the EU phytosanitary system, it lacks effective tools and protocols to successfully cope with these risks due to increased importation of plants from other continents (Eschen et al. 2017).

The overall aim of a plant health policy is to safeguard and improve the health and quality of commercially produced plants and plant products. A key element of such policy is to prevent the introduction and spread of harmful, non-native organisms and to take action through regulation of such organisms if they do become established. In Europe, based on specific pest risk analyses (PRA), about 250 plant pests and pathogens not present, or with a limited extent in the EU, are regulated (Anonymous 2016). Regulations are applied to these lists of pests or pathogens, while all the other consignments not included in the list can be introduced without any limitation. In this context, the control measures depend on the proper identification of diseases and the causal agents. Without proper identification of the disease and the disease-causing agent, disease control measures can be a waste of time and money leading to further plant losses. Proper disease diagnosis is therefore vital.

The recent history of invasions has highlighted the difficulties in halting invasive species at the border or, at least, prior to the invasion phase and preventing establishment in the EU. This is especially critical for species with long lifespans such as woody plants, which may be susceptible to many new diseases and represent potential sources of inoculum for many years. For example, *Bursaphelenchus xylophilus* (pine wilt nematode, PWN), a nematode indigenous to North America, has been introduced in Asia and, more recently, in Europe by infected timber or wood products (Vicente et al. 2012). *Xylella fastidiosa* is the agent of tracheobacteriosis, transmitted by sap-sucking spittlebug insects, and has caused a disease outbreak, affecting more than 8000 ha of olive orchards in Apulia (Southern Italy). This devastating epidemic is a significant social and economic problem in southern Italy, as well as in other European countries (Sicard et al. 2018).

Several fungal and fungal-like pathogens have been introduced into Europe and have caused considerable damage to forest and amenity tree species. The main fungal pathogens include *Cryphonectria parasitica* (causing chestnut blight), *Ophiostoma ulmi* and *Ophiostoma novo-ulmi* (Dutch elm disease), *Phytophthora cinnamomi* (Phytophthora dieback), *Ceratocystis platani* (canker stain disease of plane tree), *Seiridium cardinale* (cypress canker disease), and most recently *Phytophthora ramorum* (sudden oak death in North America and sudden larch death in Europe) and *Hymenoscyphus fraxineus* (ash dieback) (Burgess et al. 2016; Ghelardini et al. 2016; Santini et al. 2013).

Interceptions of pests and pathogens, however, probably represent only a small proportion of the alien pathogens that are arriving within the EU from other continents (Eschen et al. 2015). In particular, pathogens are difficult to detect especially when plants are asymptomatic at the time of visual inspection, and for this reason, they are spotted at lower rates respect to arthropods or nematodes (Migliorini et al. 2015). The result is their increasing establishment in natural and semi-natural ecosystems. These introduced plant pathogens coming from regions with different ecological conditions, but are sometimes able to adapt to new biotopes, and to behave aggressively on new host plants (Palm and Rossman 2003). Once introduced to new territories, they are also able to mutate, recombine, hybridize, and generate new pathogens (Fisher et al. 2012).

Furthermore, it can be expected that climate change will increase the permanent establishment of alien pathogens throughout Europe (Hellman et al. 2008). Over recent years, the consequences of climate shifts are putting ecosystems under stress, as plants do not have sufficient time for adaptation mechanisms to cope with such rapid changes. The likely increase in mean temperatures and changes in precipitation regimes will also interact with pathogen behavior (indigenous or alien), giving pathogens the opportunity to expand in areas where environmental factors previously prevented their introduction (Bergot et al. 2004; Fabre et al. 2011). All these changes will seriously impact host-parasite interactions at the tree, woodland/forest ecosystem and landscape levels (Santini and Ghelardini 2015).

The introduction of exotic plant pathogens combined with climate changes may result in new disease epidemics that can hamper efforts to manage these outbreaks. Biosecurity protocols for plant protection have been developed to prevent the diffusion and to assist in the eradication of invasive pathogens (Klapwijk et al. 2016; Lamarche et al. 2015). The protection of plants requires constant vigilance to prevent the accidental introduction of these exotic pests or pathogens, without disproportionately hindering trade. Quarantine measures are put in place by government authorities, in particular by National Plant Protection Organizations (NPPOs), to protect agricultural and forest production as well as the environment from pathogens originated from other parts of the globe. The implementation and enforcement of international phytosanitary agreements require strict controls of traded goods and knowledge of the phytosanitary status of one’s own country through monitoring and control plans.

For these reasons, a harmonized pool of novel technical, methodological, and conceptual solutions is needed to reduce and/or prevent entry and establishment of new diseases in Europe alongside mitigating the impact of invasive and indigenous organisms in agriculture and forestry, in terms of impacts on both ecosystem biodiversity and productivity. Considering prevention to be the best strategy to protect plants from diseases, in this review, we provide an overview of the new molecular-based, rapid, sharp, and reliable methods capable of intercepting woody plant pathogens before symptoms occur in the host. Based on our experience, we also suggest some advice on future requirements to prevent the introduction and spread of quarantine plant pathogens.

## Classical diagnostic methods for fungal pathogens

Some plant disease agents can be recognized from symptoms or signs determined on infected tissues (e.g., mildew on leaves, fruitbodies) by a skilled observer. There are many diseases where symptoms cannot be distinguished visually one from another, resulting in complications in the diagnosis of the pathogen. For these reasons, additional procedures for detection are needed to identify the causal agent of disease. In many cases, where the presence of a specific microorganism is unknown, the isolation and the morphological or molecular identification of the pathogen are still preferred.

Isolation of fungal pathogens from plants is usually performed by placing small portion of infected tissue on agarized growing media (Agrios 2005). In a complex natural environment, such as plant tissues, the pathogenic fungi represent a distinct minority amidst a myriad of diversified microorganisms that rapidly colonize the infected host (Tsao 1970). Despite the use of selective media, the isolation of pathogenic fungi is sometimes difficult due to the preponderance of unwanted and antagonistic fungi or bacteria, which rapidly overgrow the pathogenic fungi on the isolation plate (Catal et al. 2001). Axenic cultures can be identified by using morphological or molecular characters. In the first case, the characteristic fruitbodies of the fungus (conidia and spores) are analyzed using light microscopy: the identification of pathogen by traditional approaches is time-consuming and requires particular skills from the operator. For this reason, the identification of fungal mycelium is commonly improved by molecular markers after DNA isolation from axenic cultures.

To speed up the identification of plant pathogens and allow their identification in field, a number of serological methods have been developed, mainly based upon the enzyme-linked immunosorbent assay (ELISA). These methods are used to detect pathogens using a monoclonal antibody labeled with fluorescent compounds (Halk and De Boer 1985; Torrance 1995). There are different immunoassay methods related to visualization of the binding of a specific antibody to its related antigen (Miller and Martin 1998). The ELISA test can be easily replicated and involves the detection of a specific analyte in a liquid sample. The main disadvantage is that this method requires laboratory facilities. To overcome such limitations, portable immunoassay methods were developed. Lateral flow devices (LFDs) are a simple paper-based dip-stick assay to detect the presence or absence of a target analyte in a liquid sample without the need for specialized laboratory equipment. This method has become widespread over the last few years allowing rapid in-field detection of plant pathogens (Boonham et al. 2008; Tomlinson et al. 2010). The LFD method is simple to use and is able to detect and identify the causal agents of disease in few minutes.

However, the immunological methods require the availability of an antibody that properly responds to a target pathogen. In addition, incorrect diagnosis may occur due to the presence of a false positive resulting from a nonspecific antibody-antigen reaction. Serological techniques applied to the detection of phytopathogenic fungi have not been as successful as in bacteriology or virology, largely due to the high variability and phenotypic serological plasticity of fungi. Despite several portable kits being commercialized for different pathogens, the immunological methods are generally less sensitive than molecular methods. For these reasons, the plant pathology detection techniques have moved towards faster and more sensitive approaches, such as molecular methods.

## History of molecular methods for fungal pathogens of woody plants

Over the last three decades, diagnostics in plant pathology have undergone important changes. In the early years, plant pathology laboratories primarily developed methods to analyze genetic differences among fungal populations. In the 1990s, a reliable method for taxonomic characterization of fungal isolates was based on electrophoretic examination of mycelia extracellular enzymes. These approaches allowed the use of allozyme and isozyme markers to rapidly differentiate intersterility groups of *Heterobasidion annosum* (Goggioli et al. 1998; Karlsson and Stenlid 1991; Otrosina et al. 1992), *Phytophthora cinnamomi*, and *Seiridium* sp. isolates (Old et al. 1984; Raddi et al. 1994). These techniques require prior isolation of the pathogen in pure culture and are therefore poorly adapted to direct *in planta* detection.

**Fig. 1:**
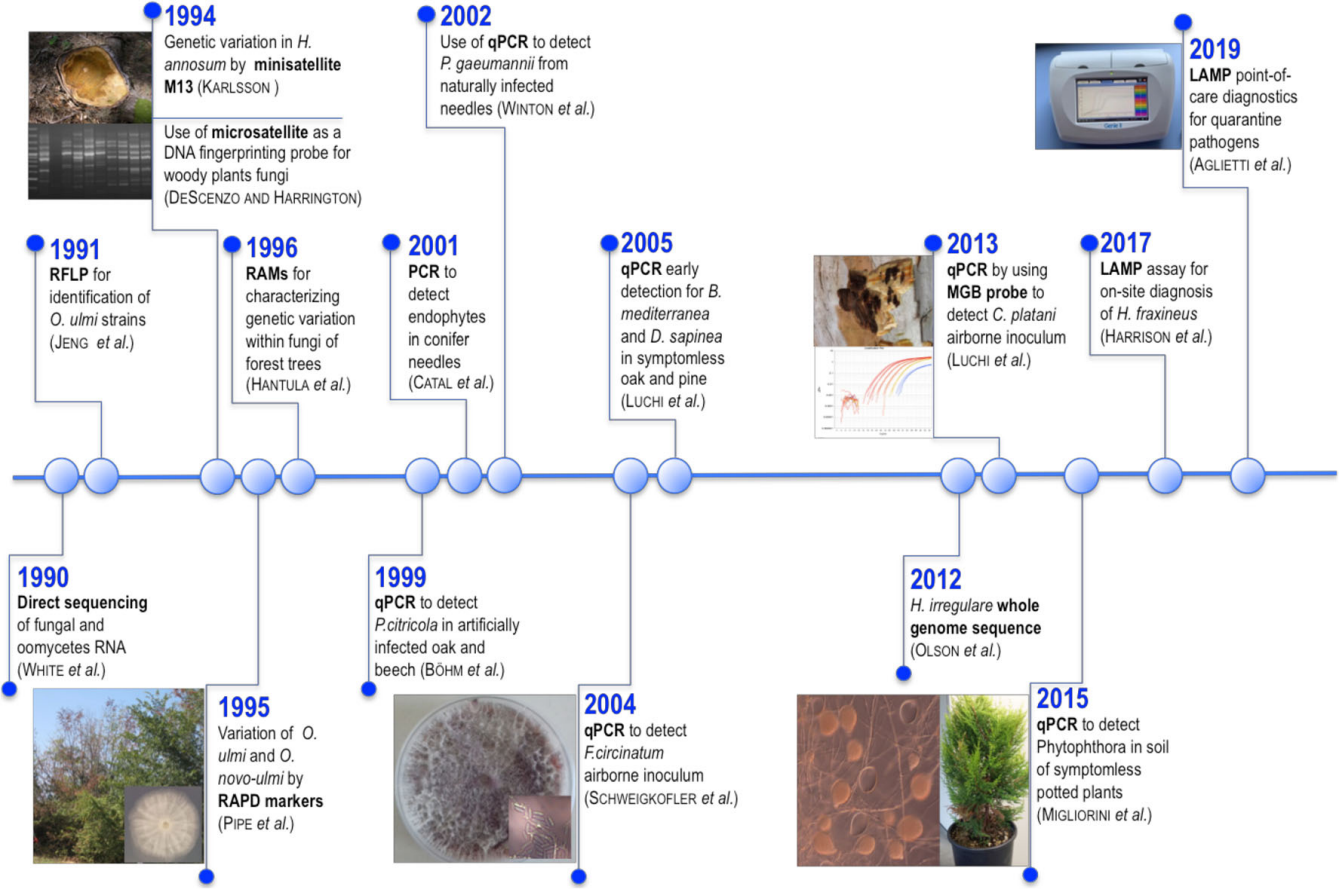
Timeline of molecular detection methods in woody plant pathogens

Over the same period, diagnostic techniques based on nucleic acid markers were becoming more established: detection tools and methods to identify fungal species were being progressively influenced by advances in molecular biology (Fig. 1). The discovery of PCR (polymerase chain reaction) revolutionized molecular diagnostics, allowing characterization of fungal pathogens by the direct sequencing of ribosomal RNA genes (White et al. 1990). Multiple technologies, based on specific DNA-based markers, have been developed both for fungal diagnostics and population studies. These include restriction fragment length polymorphism (RFLP) initially used to study genetic diversity in *Ophiostoma ulmi* (Jeng et al. 1991; Hintz et al. 1991) and *Ceratocystis* species (Witthuhn et al. 1999) (Fig. 1). DNA-fingerprinting based on microsatellite and the minisatellite DNA markers was then applied to different forest pathogens (DeScenzo and Harrington 1994; Karlsson 1994; Santini et al. 2005; Santini and Capretti 2000; Stenlid et al. 1994; Vasiliauskas and Stenlid 1997) (Fig. 1).

Random amplification of polymorphic DNA (RAPD) markers was also used to assess variation of *O*. *ulmi* and *O*. *novo-ulmi* isolates (Pipe et al. 1995) and other woody fungal pathogen populations (Goggioli et al. 1998; Halmschlager et al. 1995; Hansson et al. 1996; Zhou and Stanosz 2001).

In 1996, a novel method, random amplified microsatellite (RAMS), was developed to generate DNA markers in higher fungi (Hantula et al. 1996) (Fig. 1). This method became widely used for population studies. Later on, both RAPD and RAM PCR fragments were used as targets for the design of specific target sequence characterized amplified region (SCAR) primers (D’Amico et al. 2007). These genomic regions generated by random DNA amplification have proven to be particularly appropriate for species identification in situations when housekeeping genes used for phylogeny were not sufficiently discriminating. Moreover, SCAR was successfully used to detect hybrid-specific regions in hybrid pathogens such as for *Phytophthora alni* (Ioos et al. 2005).

In the early 2000s, studies on fungal DNA detection rapidly moved to detect fungal pathogens in symptomless plant tissue. Catal et al. (2001) developed the first PCR to quantify and identify endophytes in symptomless conifer foliage (Fig. 1). Technical improvements in the amplification of nucleic acid target allowed the development of a new PCR capable of detecting and quantifying results in real-time (quantitative real-time PCR or qPCR). The introduction of qPCR (Livak et al. 1995) allowed the quantification of nucleic acids with higher sensitivity and specificity due to the real-time monitoring of the amplification reaction and the use of a third-level specific reagent: the probe. The fluorescence that is monitored during the entire qPCR process can be detected by sequence-specific fluorescent oligonucleotide probes (i.e., hydrolysis or scorpion probes, or molecular beacons), or by a nonspecific detection strategy independent of the target sequence, e.g., through fluorescent dyes that have special fluorescent properties when bound to dsDNA (i.e., SYBR Green) (Gachon et al. 2004). The use of fluorescence as the detection signal improved the robustness of the system and its consequent applicability using automated devices.

The first studies on the use of qPCR in forest pathology were conducted to detect *Phytophthora citricola* in oak and beech (Böhm et al. 1999) and *Phaeocryptopus gaeumannii* in Douglas Fir (Winton et al. 2002) (Fig. 1). During the years, sensitivity and specificity of qPCR allowed its application to detect fungal pathogens directly from samples extracted from infected symptomatic and asymptomatic tissue, including seeds, or samples where pathogens were difficult to isolate, i.e., airborne fungal inoculum (Fig. 1; Table 1).

**Table 1:** Examples of alien and native expanding pathogens for EU for whom a molecular detection tool has been developed

Pathogen	Associated disease	Host range	Present distribution^1^	Status	First report (year)	Spread	Impact	Detection method^2^	Reference
*Biscogniauxia mediterranea*	Charcoaldisease	*Quercus* spp.	EU	Native	-	Air	Medium	qPCR	Luchi et al. (2005a)
*Biscogniauxia nummularia*	Charcoaldisease	*Fagus sylvatica*	EU	Native	-	Air	Medium	qPCR	Luchi et al. (2006)
*Caliciopsis pinea*	Caliciopsiscanker	*Pinus* spp*.*	EU, USA	Cryptogenic	-	Air	Medium/High	qPCR	Luchi et al. (2018)
*Ceratocystis platani*	Canker staindisease	*Platanus* spp.	EU, TR,USA	Alien	1971	Human	High	qPCR, LAMP	Aglietti et al.(2019a); Luchi et al. (2013)
*Diplodiasapinea*	Shoot blight	*Pinus* spp.*Pseudotsugamenziesii*	EU, USA,ZA	Native	-	Air, insects	High	qPCR, HRMA	Luchi et al. (2005b, 2011)
*Diplodiascrobiculata*	Shoot blight	*Pinus* spp.	USA, ZA	Alien	Notpresent	Air	Medium	HRMA	Luchi et al. (2011)
*Dothistromapini*	Red bandneedleblight	*Pinus* spp.	USA, EU	Alien/Native	-	Air	High	qPCR	Ioos et al. (2010)
*Fusariumcircinatum*	Pine pitchcanker	*Pinus* spp.	EU, USA, ZA, CL	Alien	1995	Air	High	qPCR, LAMP	Aglietti et al. (2019b); Grosdidier et al. (2017);Ioos et al. (2009a, 2019a); Luchi et al.(2018); Schweigkofler et al. (2004)
*Fusariumewallaceae*	Fusarium wilt	Broad range	IL, USA, ZA	Alien	Not present	Insects	High	qPCR,LAMP	Aglietti et al. (2019b)
*Heterobasidionirregulare*	Irregulare root disease	*Pinus* spp.	EU	Alien	2004	Air	High	LAMP	Sillo et al. (2018)
*Heterobasidionspp.* (incl*. H.irregulare*)	Root rot	Conifers	Global	Alien/Native	-	Air	High	qPCR	Ioos et al. (2019b)
*Hymenoscyphusfraxineus*	Ash dieback	*Fraxinus* spp.	EU	Alien	1992	Air	High	qPCR	Chandelier et al.(2010);Grosdidier et al. (2017); Ioos et al. (2009b)
*Melampsoramedusae* f.sp.*deltoidae*	Rust	Poplars	NA, SA,ZA, OC,AS, EU	Alien	2018	Air	Low	PCR, qPCR	Boutigny et al. (2013a);Husson et al. (2013)
*Melampsoramedusae* s.l.	Rust	Poplars	NA, SA,ZA, OC AS, EU	Alien	1993	Air	Low	qPCR	Boutigny et al. (2013b)
*Phytophthoraalni*	Alderdieback	*Alnus* spp.	EU	Alien	1994	Water	High	PCR,qPCR	Husson et al.(2015); Ioos et al.(2005)
*Phytophthoralateralis*	Port-Orford-Cedarroot disease	Chamaecyparis,Taxus, Thuja	NA, EU, TW	Alien	2004	Air, water	Moderate	qPCR	Schenck et al.(2016)
*Phytophthoraramorum*	Sudden oakdeath;Suddenlarchdeath	Broad range	EU, USA	Alien	1995	Air,water	High	qPCR,LAMP	Aglietti et al.(2019a); Iooset al. (2006); Migliorini et al. (2019)
*Phytophthora*spp.	Phytophthorablight	Broad range	Global	Alien/Native	-	Water(mostly)	High	qPCR	Migliorini et al. (2015, 2019)

^1^AS=Asia; CL=Chile; EU=Europe; IL=Israel; NA=North America; OC=Oceania; SA=South America; TR=Turkey; TW=Taiwan; USA= United States of America; ZA=South Africa.

^2^*HRMA*, High Resolution Melting Analysis; *LAMP*, Loop-mediated isothermal amplification; *PCR*, Polymerase chain reaction; *qPCR*, Real-time quantitative PCR.

Improvement in molecular techniques allowed a rapid alternative to rDNA sequencing: whole genome sequencing. In 2012, the forest pathogen and wood decayer *Heterobasidion irregulare* was fully sequenced with standard Sanger sequencing protocols by using three different sized libraries (Olson et al. 2012). However, this method, requiring a prior step of isolation of the pathogen in pure culture, is too expensive and too slow for a routine use. In recent years, “*democratization*” of high-throughput sequencing technologies (454-pyrosequencing, Illumina MiSeq, PacBio, Nanopore, etc.) and their potential for identification have been exploited to amplify in bulk the same region (barcode) of the genome from all the microorganisms present in an environmental sample (metabarcoding). With these techniques, it is not necessary to isolate each microorganism beforehand and analyze it individually, since the set of barcodes is amplified in one single reaction. The huge amount of DNA barcodes generated must then be analyzed by bioinformatic processing to clean, sort, cluster, and compare the amplified sequences with reference databases to finally produce an inventory of the biodiversity present in a sample, including pathogens. This non-targeted molecular technique may be used to identify a known forest pathogen, particularly a quarantine organism, by comparing its barcode with reference databases (Aguayo et al. 2018; Bulman et al. 2018; Català et al. 2016; Chen et al. 2018). An additional advantage is that they also make it possible to identify an unknown pathogen, which does not match with any reference sequence in the databases. Nonetheless, additional investigations, such as pathogenicity tests, will be required to assess the threat posed by these “unknown” pathogens.

Despite the growing development of molecular techniques, the need to use rapid and sensitive techniques capable of detecting a pathogen before its spread has even become evidently increasing. More recently, the growing need for field instrumentation has led to the development of portable methods based on isothermal amplification (Fig. 1). Loop-mediated isothermal amplification (LAMP) of DNA is a newer molecular technology for affordable, specific, highly sensitive, and rapid diagnostic testing of pathogens in both laboratory and field conditions. The LAMP technique involves the optical excitation and detection of a pathogen’s DNA in an environmental sample mixed with a fluorescent dye as it is heated and amplified in less than an hour. This method has been described by Notomi et al. (2000) and subsequently been optimized for portable instruments in field. Recently several protocols for a rapid detection of woody pathogens, such as *C*. *platani*, *F*. *circinatum*, *F*. *euwallaceae*, *H*. *fraxineus*, and *P*. *ramorum*, have been established (Aglietti et al. 2019a, 2019b; Harrison et al. 2017) (Table 1).

## Development and validation of molecular diagnostic methods

**Fig. 2:**
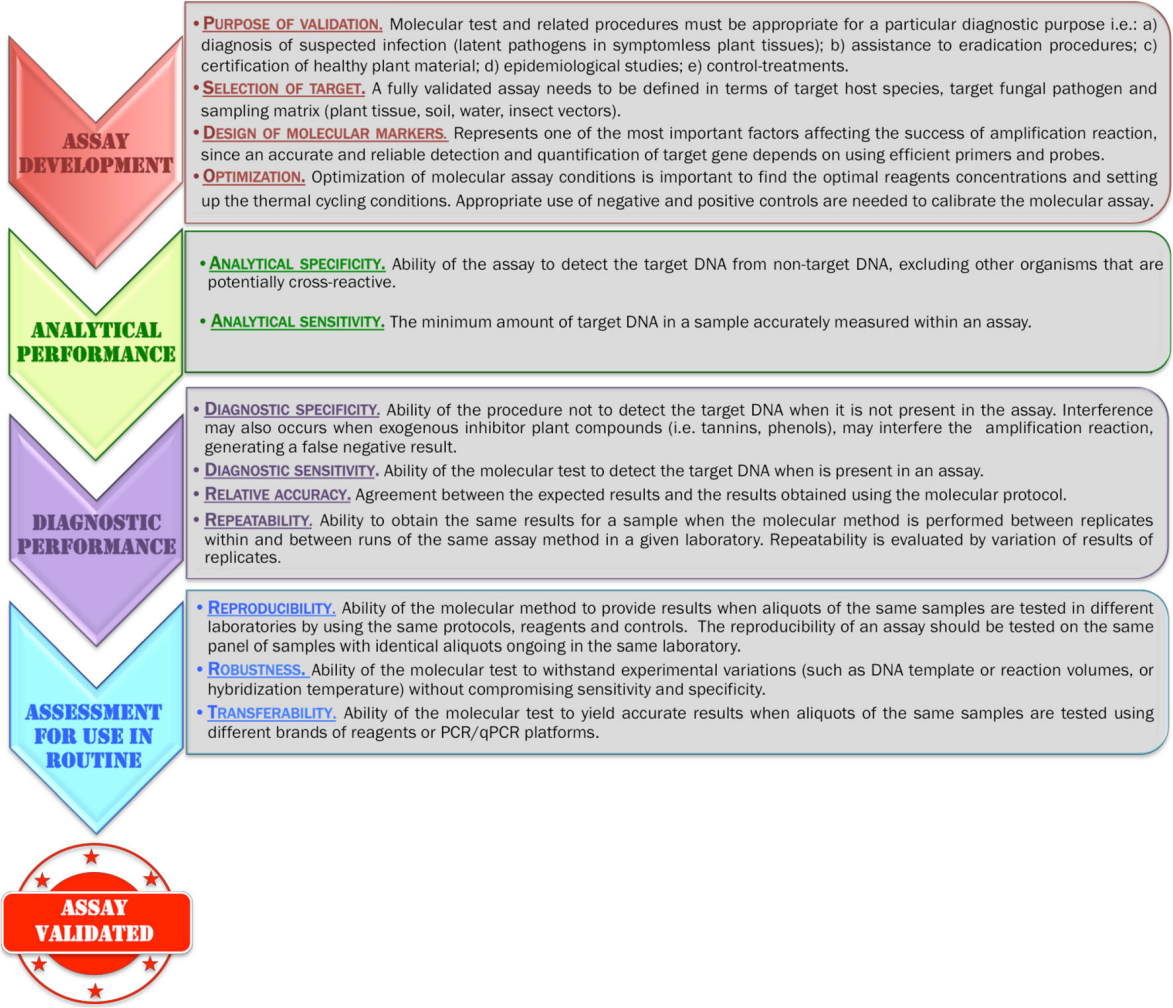
Molecular assay development and method validation

Historically, diagnosis and identification in mycology were based on direct observation of macro- and microscopic structures of fungi. The image was, and remains, a tangible proof of the existence of the microorganism. However, the lack of resolution and sensitivity of this type of approach, and it must be emphasized, and the loss of skills in fungal morphological characterization have oriented laboratories towards these new molecular technologies that are increasingly standardized and theoretically more objective. Most of the new molecular diagnostic methods produce results in the form of signals (florescence, pH, electricity, etc.) derived from the presence of targets (nucleic acids). These signals are very weak and must be multiplied and amplified to be detectable by a device or by human eye interpretation. They are therefore inherently subject to errors, contamination, and drift and remain indirect evidence of the presence of a microorganism. The validation and implementation of these technologies are in theory and in practice extremely sensitive and definitive, and must therefore be performed with a minimum of precautions and controls. Validation of the diagnostic method is a procedure that assesses the performance of a molecular assay, which has been developed for a specific purpose. As a preliminary to the development of a new detection test, it is therefore necessary to define upstream the needs in terms of the minimum level of specificity, sensitivity, cost, speed of execution, and any other criteria that will allow assessment of the test being adapted in relation to the pursued objective. Molecular diagnostic assays must be validated for the target pathogen both in the laboratory and eventually in the field, following different steps: (a) assay development; (b) analytical performance; (c) diagnostic performance; (d) reproducibility, transferability, and robustness (Fig. 2).

The first step is the assay development where the purpose of validation and the target host species, fungal pathogen, and sampling matrix are defined (Fig. 2). The main challenge of molecular approaches is the rapid screening of a target DNA sequence. Over recent years, different molecular methods have been developed for the detection and identification of different taxonomic groups based on “barcode” sequences. These approaches are based on the hypothesis that each species carries specific DNA sequences that are different from those found in individuals from other species. However, DNA sequences may exhibit variation within species that can be manifested by the insertion or deletion of a single nucleotide and are strictly related to the ecology of the individual (i.e., reproductive success, migration, genetic drift) (Möller and Stukenbrock 2017). In this respect, for a group of pathogens as complex and polymorphic as fungi and oomycetes, genetic variability may affect the reliability of a test based on specific recognition and amplification of nucleotide sequences by affecting the sequence of the regions targeted by oligonucleotides (PCR primers and probes) (Vincelli and Tisserat 2008). As a result, the DNA of target organisms can therefore no longer be detected, or conversely, that of non-target organisms can be erroneously detected.

The success of molecular identification is strictly related to the choice of the target gene, accounting for possible intraspecific and intraspecific variation to avoid overlap with other individuals and species. For this aim, the ribosomal RNA internal transcriber spacer (ITS) sequence is the gene more widely used and generally considered as the “barcode” sequence (Schoch et al. 2012).

Nowadays, with a wide availability of molecular techniques, it has become clear that fungal taxonomy is more complicated than expected and sometimes presents some conflicts in the definition of different taxonomic entities (Wingfield et al. 2011).

The use of a single molecular marker to identify a target microorganism could be inappropriate because of the presence of new cryptic species or the re-assignation of species into new taxa. For example, in the Botryosphaeriaceae family, to characterize and distinguish strictly phylogenetically related species, a multigene analysis using 3–4 different genes is strongly needed (Fourrier et al. 2015; Slippers et al. 2013), even if using high-resolution melting analysis (HRMA) makes it possible to distinguish three closely related species of *Diplodia* (Luchi et al. 2011).

In this context, the assignment of a microorganism to a meaningful category needs to be well considered by both molecular and morphological characters. A stable classification and appropriate nomenclature of target pathogens are therefore crucial to design specific molecular markers that then need to be validated. In this respect, the development of detection tests requires a thorough preliminary study of the genetic diversity of the target species and closely related taxa.

The “*democratization*” of genome sequencing opens new and powerful tools for screening polymorphisms and divergent regions, which can then potentially be exploited for the design of specific oligonucleotides. Comparing whole genomes of fungi for the purpose of finding polymorphous regions can be done in different ways. Some studies have proposed algorithms to identify single-copy gene homologs encoding proteins (Aguileta et al. 2008; Feau et al. 2011; Marthey et al. 2008). These algorithms were initially used to search for new phylogenetic markers that are more discriminating than the traditional markers, and could therefore be targeted as taxonomic markers for molecular detection tools. Some teams have already used these new molecular markers from orthologous gene bases to define degenerate primers to obtain the sequence of these molecular markers and confirm their utility for robust phylogenetic studies (Schmitt et al. 2009; Vialle et al. 2013).

To assess the best performance of the molecular method optimization is needed through adjustment of the most important parameters of the assay, such as analytical specificity, analytical sensitivity, selectivity, reproducibility, and repeatability performance characteristics (EPPO 2014) (Fig. 2). Once primer and probe concentrations are validated, the correct calibration of the assay is tested by a standard curve and a melting curve analysis (Bustin and Huggett 2017). During the calibration of the molecular assay, the appropriate use of control is important. The negative control includes all amplification reagents but no DNA and is used to guarantee that reagents are free of contamination or that they are not included during the amplification. The positive control includes a representative sample of the target DNA to be detected and is used to ensure no problems with the amplification reaction whether it be from the reaction components or the instrument itself. Reference samples are routinely included as control for the assay, providing monitoring of the method. For this reason, particular attention must be paid to the storage of the reference DNA to ensure its stability (Bustin et al. 2010).

Once the assay has been validated, the analytical performance is determined by testing the analytical specificity and sensitivity (Fig. 2). The analytical specificity is performed by testing a broad species of the same taxon, including those from different geographical locations. Moreover, also out-group species DNA need to be tested to avoid erroneous identification of target pathogens. The measure of the analytical sensitivity is the limit of detection (LOD) determined by a serial dilution of target DNA and the final dilution showing consistent positive response is considered to present the limit of detection. Diagnostic performance is evaluated by different indicators (Fig. 2) and then the reproducibility, robustness, and transferability confirm the reliability of the test when performed in different laboratories. Reproducibility corresponds to the ability of the test to yield accurate results when carried out with different operators and equipment, whereas robustness reflects its ability to withstand slight changes in experimental conditions with respect to a standard protocol (Vander Heyden et al. 2001). Transferability is an equally important parameter for validation. Although a test is sometimes very efficient in the initial conditions of development, it is conceivable to imagine that the performance of a qPCR or PCR detection protocol may vary if the conditions of use differ from those of the development team (Grosdidier et al. 2017). In a study focused on the transferability of molecular test detection, in the pine pathogen *Fusarium circinatum*, Ioos et al. (2019a) showed that changes in reagents, equipment, and qPCR fluorescence data processing software could have major effects on the reliability of the results (false positives and false negatives). Much of the variance produced in qPCR results (expressed as Ct values) is not simply due to reagent changes. It is also caused by the corrections applied by the fluorescence data processing algorithms, each of which manually or automatically makes its own assumptions about the measured data.

Once the last stage has been completed, assuming that the previous stages have been satisfactory, the molecular test can be designated as valid for the originally intended purpose (Fig. 2).

## Challenges of diagnostic methods: a continuous diagnostic urgency for plant health

The time needed to diagnose plays a relevant role in the prevention of pathogens spreading, and also the correct management of the outbreak of a disease. The challenges of molecular diagnostics are focused around the need to rapidly and accurately identify the causal agent of plant disease directly within host plant or different substrates (i.e., soil, water, airborne). In recent years, a variety of excitable fluorescent dyes have been widely used to monitor the amplification process in real-time, representing a significant advance in many molecular techniques involving detection of nucleic acids. The detection of PCR products by way of a fluorescent dye can be quantified using a qPCR thermal cycler or a portable fluorescent reader, which is equipped with a battery pack for rapid onsite detection (Hughes et al. 2006; Tomlinson et al. 2005). Based on this approach, both of the main techniques, i.e., qPCR and LAMP assays, enable rapid and specific detection of pathogens from environmental samples (Table 1).

**Fig. 3:**
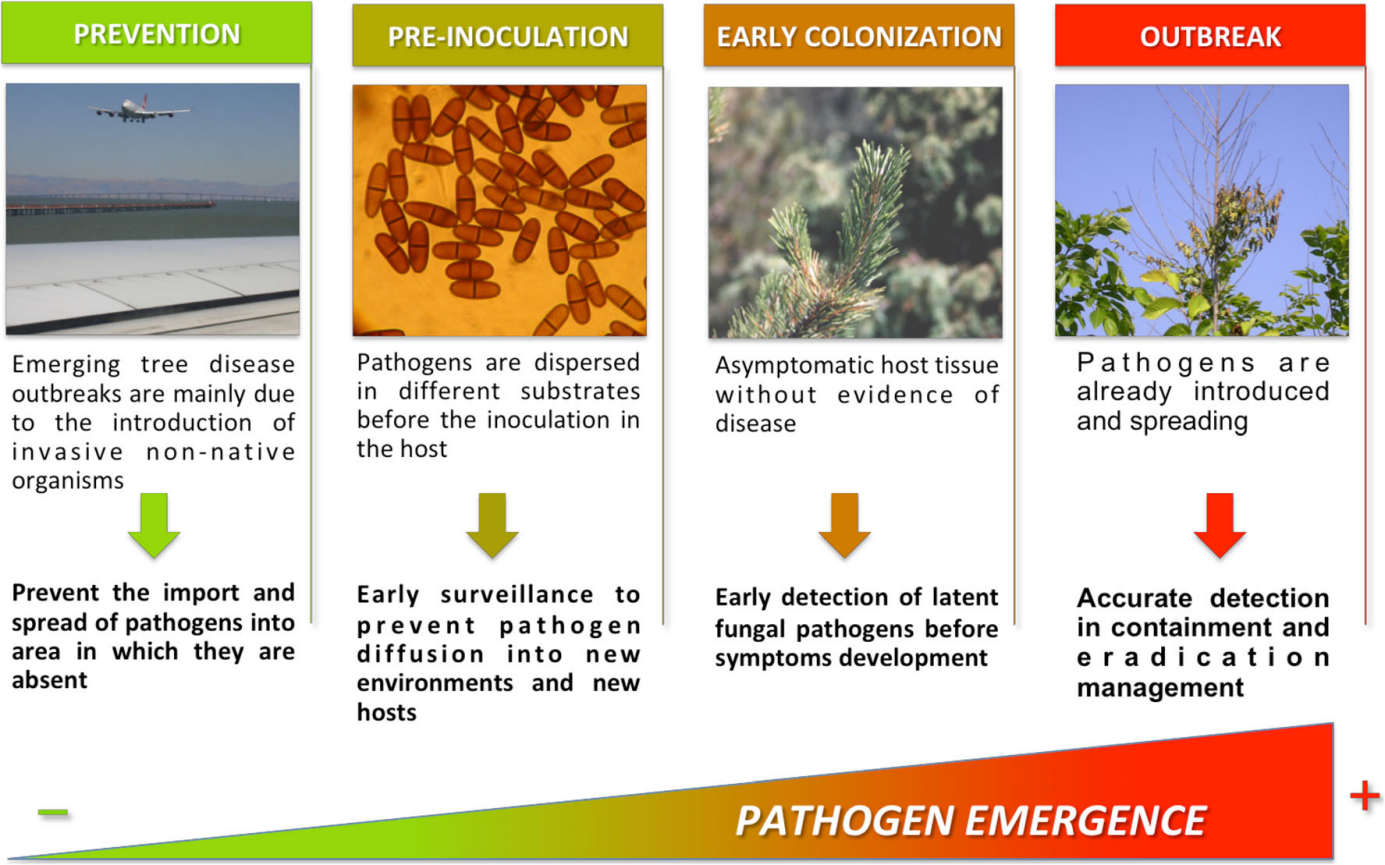
Application of molecular techniques for plant pathogen detection in different steps of the invasion process

Due to the speed, reliability, and sensitivity of these innovative approaches, they can be used for prevention and control of disease (Fig. 3).

### Disease prevention

Emerging tree disease outbreaks are mainly due to the introduction of invasive non-native organisms or the emergence of a native organism following changes in climatic conditions or silvicultural management. New non-native fungal pathogens of trees are establishing at increasing rates in many parts of the world. This process goes alongside the rapid increase in the volume and diversity of intercontinental trade in plants, which is the main pathway for introduction of insects and fungi, as contaminants and hitchhikers, as well as wood and wood products (Chapman et al. 2017; Liebhold et al. 2012; Santini et al. 2013, 2018). The current methods for preventing the introduction of invasive pathogens consist mainly in the visual assessment of the aerial parts of the plant for symptoms, or of plant products on a small number of economically important plant pests reported on quarantine lists. Once the pathogen is established then eradication is the only option, even if it is often unsuccessful and more expensive. The application of rapid detection tools as point-of-care diagnostics and for the reinforcement of border biosecurity is highly desirable, reducing the detection time to intercept quarantine species. Significant advantages could be gained by relocating testing technology closer to the site of sampling. This is the case of LAMP portable assays. A number of different assays have been developed for the early detection of woody pathogens, including quarantine species (*P*. *ramorum*, *C*. *platani*, *H*. *fraxineus*, *F*. *circinatum*, and *F*. *euwallaceae*) (Aglietti et al. 2019a, b; Harrison et al. 2017).

Most of the epidemics that occurred during last decades were caused by alien pathogenic organisms previously unknown to science or not known to be pathogens. To address this gap in our knowledge, one of the most challenging fields in plant health is the identification of potential threats before their introduction in a new environment. The trade in forest tree seeds is therefore considered to be high risk for the introduction of fungal pathogen in disease-free areas (Burgess and Wingfield 2002; Franić et al. 2019), and sensitive tests are required to check their health status before movement (Ioos et al. 2009a; Lamarche et al. 2015). Sentinel planting, i.e., plants native to importing country planted in exporting countries that are inspected and analyzed at regular intervals for microbial pathogen detection, is the most promising tool (Eschen et al. 2019). To this end, one could imagine associating other types of “biological” sensors that have already been the subject of promising studies, such as the sentinel plantations of European species exposed in an exotic environment (Vettraino et al. 2015) or sentinel nurseries of non-native species found in their non-native environment of origin (Vettraino et al. 2017). This type of sentinel also makes it possible to study the pathogenic potential and the phytosanitary risk caused by these taxa identified by their bar code sequence in air trapping (Aguayo et al. 2018). Once the potential threat is identified, it is then possible to develop molecular tools to prevent its introduction in the importing country, and portable diagnosis devices represent a perfect tool to reinforce border security and prevent new introductions.

### Disease control

Once a pathogen is present in the environment, the disease can be monitored at different stages (Fig. 3).

#### Pre-inoculation

Before arriving in the host, pathogen inoculum is dispersed in different substrates (soil, water, air, and insects). For example, the incubation period of soilborne plant pathogens, which are difficult to detect as they spread and infect the host underground, could persist for long periods with aboveground symptoms occurring even later. Moreover, detection of soilborne pathogens could be affected by soil conditions, including the complex resident microflora, making diagnosis difficult. For these reasons, the development of molecular methods to detect inoculum of the pathogens in soil and water is of a primary importance. Migliorini et al. (2015) developed a qPCR able to detect small amounts of *Phytophthora* DNA inoculum in samples of soil and irrigation water in nurseries.

Pathogens are also dispersed by airborne inoculum, which can vary in time and space.

New opportunities to combine air sampling with qPCR to identify and quantify fungal pathogens have been developed for *Caliciopsis pinea*, *C*. *platani*, *F*. *circinatum*, *H*. *fraxineus*, and *Phytophthora* spp. (Botella et al. 2019; Chandelier et al. 2010, 2014; Luchi et al. 2013; Migliorini et al. 2019; Schweigkofler et al. 2004). The use of these techniques can provide more accurate forecasts of the risk of pathogen spread, and help the management of the disease. This is the case in *C*. *platani*, where airborne inoculum of the pathogen was found within 200 m of the closest symptomatic infected plane tree and in the surrounding area with healthy plane trees (Luchi et al. 2013). Grosdidier et al. (2018) investigated the spatial and seasonal dispersal of *H*. *fraxineus* airborne inoculum in France and found that, during the sporulation peak, spores were detected up to 50–100 km ahead of the disease front, showing the presence of the pathogen before any visible symptoms. For these reasons, the development of highly sensitive and reliable molecular detection methods could help prevent the invasion of pathogen in uncontaminated areas, improving the activity of the National Plant Protection Organizations (NPPO) by shaping more efficient disease management strategies.

Insects play an important role in vectoring tree pathogens and in their establishment and reproduction in the host (Kirisits 2004). The use of molecular tools, such as those based on qPCR and LAMP assays, significantly improves biological and ecological studies of insect-fungus interactions. The qPCR was able to ascertain the association between the western conifer seed bug *Leptoglossus occidentalis* and the fungus *Diplodia sapinea* that causes damages on pine cones in Mediterranean forests (Luchi et al. 2012). Villari et al. (2013) developed a LAMP assay to rapidly detect blue stain fungus directly from bark beetles, while Fourrier et al. (2015) designed a qPCR assay to detect *F*. *circinatum* from collected bark beetles, allowing to monitor the spread of the quarantine pathogen across different geographical areas.

#### Early colonization (asymptomatic phase)

Some fungal species have the ability to survive in a latent phase within their hosts, causing disease when the host is exposed to environmental stress (Coutinho et al. 2007; Desprez-Loustau et al. 2006; Slippers et al. 2017; Slippers and Wingfield 2007; Smith et al. 1996; Stanosz et al. 2001). In this case, the development and standardization of rapid diagnostic assays, with adequate sensitivity and specificities, would aid in determining the presence of the pathogen at the beginning of infection. For the detection of pathogenic fungi in asymptomatic woody tissues, an appropriate sampling technique should be considered and associated with the detection protocol, as it will largely determine its sensitivity. The qPCR represents the gold standard for the quantitative measurement of nucleic acids and is thus the more appropriate tool for the early detection and quantification of latent invaders (Luchi et al. 2016, 2020). The sensitivity of this approach has been developed to detect low amount of the DNA of fungal pathogens, which could become harmful to trees stressed by abiotic factors as water deficiency (Luchi et al. 2005a, 2005b, 2006). Maresi et al. (2007) found that the incidence of *D*. *sapinea* in Austrian pine in its asymptomatic phase was strongly related to some environmental parameters. These results confirm that qPCR is a powerful tool to improve the evaluation and prevention of sanitary risks by providing an opportunity to study the spatial and temporal dynamics of diseases in field.

In the future, it seems equally important to have tools and, above all, a sampling method to obtain data regarding the prevalence of host plants that are infected in a cryptic manner and therefore asymptomatic but potentially infectious (Luchi et al. 2016; Thompson et al. 2016). As already demonstrated for *F*. *circinatum* (Storer et al. 1998; Coutinho et al. 2007), infection with many phytopathogenic fungi may remain cryptic until environmental conditions or stress induce the switch to the pathogenic form (Agrios 2005). This seems particularly relevant for phytopathogenic fungi and oomycetes affecting woody species, which require long periods of incubation and latency before disease symptoms become visible and infectious (Vicent and Blasco 2017). Such asymptomatic infections with *D*. *sapinea* have already been demonstrated by qPCR on pines (Maresi et al. 2007). Latent infections with several polyphagous *Phytophthora* species have also been demonstrated on a large scale in nurseries (Migliorini et al. 2015). Several examples have been documented in the genus *Fusarium*. *Fusarium circinatum*, the pitch pine canker agent, has been isolated from many herbaceous plants near infected patches, in a form that appears to be endophytic, although at intact pathogenicity when inoculated on pine (Swett and Gordon 2012; Swett et al. 2014).

The detection and quantification of specific weakness pathogens in symptomless host tissues represent a useful tool to evaluate the general physiological status of a single tree and tree communities and to forecast the outbreak of complex decline syndromes.

#### Outbreak (symptomatic phase)

A key parameter for the early assessment of the size of an outbreak is the sensitivity of the surveillance methods. In this context, the use of the previously described molecular techniques proved to also be useful in constraining the spreading of already introduced pathogens and exploring the epidemiological dynamics of disease. Sensitive and reliable real-time PCR assay was developed to detect *P*. *ramorum* from hundreds of symptomatic hosts collected from different sites in California (Hayden et al. 2004). Tooley et al. (2006) developed a multiplex assay to detect *P*. *ramorum* and *P*. *pseudosyringae* (a species that causes symptoms similar to *P*. *ramorum*) in field samples in California. The use of a multiplex assay allowed the simultaneous detection of different pathogens in the same PCR reactions. A multiplex quantitative PCR to simultaneously test total needle DNA for the presence of *Dothistroma septosporum*, *D*. *pini*, or *Lecanosticta acicola* has been developed (Ioos et al. 2010; Schneider et al. 2019). The use of a multiplex assay is particularly important to distinguish pathogens that cause similar symptoms. A new PCR assay has been developed to also distinguish *Fusarium circinatum* and *Caliciopsis pinea* that cause comparable symptoms on *P*. *radiata* in the initial stages of colonization (Luchi et al. 2018). All four European species of *Heterobasidion* attacking conifers may be simultaneously detected in two reactions (Ioos et al. 2019b).

Changes in the evolutionary behavior of fungal pathogens might be also triggered by climate change inducing incidence of new disease outbreaks on new hosts in new geographical area. This is the case of *D*. *sapinea* recently found on *Pinus sylvestris* in north Europe (Brodde et al. 2019), as well as the occurrence of new hosts in previously unaffected northern hemisphere countries and for *Dothistroma septosporum* and *D*. *pini* (Mullett et al. 2018). The use of molecular tools may assist in the monitoring of disease spread in extensive monitoring surveys. Oak et al. (2008) used nested or qPCR to detect the presence of *P*. *ramorum* (between 2003 and 2006) in more than 12,000 samples from 44 hosts in the US forests. More recently, Heller and Keith (2018) developed a qPCR assay for monitoring and managing Rapid Ohia Death (ROD) pathogens caused by *Ceratocystis lukuohia* and *C*. *huliohia* in Hawaiian Islands.

Moreover, a significant improvement in monitoring systems will be the use of portable devices for rapid and on-site pathogen diagnosis such as the specific diagnostic LAMP assays for *H*. *irregulare* and *C*. *platani* by analyzing sawdust collected from infected trees (Aglietti et al. 2019a; Sillo et al. 2018).

## Conclusions and future perspectives

In the past, the development of molecular diagnostic assays for plant pathogens has been labor-intensive and required a high level of technical expertise. In recent years, the number of protocols commercially developed to detect fungal pathogens has increased. This has increased interest in new molecular diagnostic tools for the identification of plant pathogens.

The rise of a variety of new molecular tools radically changed approaches in plant pathology. Fungal isolation for diagnostic aims is no longer necessary, results from molecular tools are achieved within hours instead of days, and detection protocols are more specific and sensitive. These accurate and sensitive techniques are particularly of interest for vulnerable long-living plants such as forest trees. Emerging technologies enable the cost-effective and high-throughput detection and quantification of pathogens with speed, sensitivity, and ease of use. Promising portable molecular detection systems for emerging pathogens in the field and the reinforcement of border biosecurity are now available. These technologies result in timely, accurate, and effective tools for early surveillance and management of plant diseases, representing a valuable tool for plant biosecurity.

Even if there is a continuous improvement of molecular techniques, the perfect molecular method is not yet available: all methods have both advantages and limitations. It is then recommended to use different molecular approaches to achieve rapid and safe detection of plant pathogens.

## References

[CR1] Aglietti C, Luchi N, Pepori AL, Bartolini P, Pecori F, Raio A, Capretti P, Santini A (2019). Real-time loop-mediated isothermal amplification: an early-warning tool for quarantine plant pathogen detection. AMB Express.

[CR2] Aglietti C, Stehliková D, Paap T, Luchi N, Pecori F, Santini A (2019b) A new loop mediated isothermal amplification assay based on assimilating probe for early sequence-specific detection of *Fusarium circinatum* and *F. euwallaceae*. Joint Meeting of the IUFRO Working Parties “Shoot, foliage and stem diseases” and “Wilt diseases” (7.02.02 and 7.02.03) on Phyllosphere Diseases. 6-10 May 2019, Figline Valdarno, Florence, Italy, pg 49

[CR3] Agrios GN (2005). Plant pathology.

[CR4] Aguayo J, Fourrier-Jeandel C, Husson C, Ioos R (2018). Assessment of passive traps combined with high-throughput sequencing to study airborne fungal communities. Appl Environ Microbiol.

[CR5] Aguileta G, Marthey S, Chiapello H, Lebrun M-H, Rodolphe F, Fournier E, Gendrault-Jacquemard A, Giraud T (2008). Assessing the performance of single-copy genes for recovering robust phylogenies. Syst Biol.

[CR6] Anonymous (2016) Regulation (EU) 2016/2031 on protective measures against plant pests (“Plant Health Law”)

[CR7] Bergeron MJ, Feau N, Stewart D, Tanguay P, Hamelin RC (2019). Genome-enhanced detection and identification of fungal pathogens responsible for pine and poplar rust diseases. PLoS One.

[CR8] Bergot M, Cloppet E, Pérarnaud V, Déqué M, Marçais B, Desprez-Loustau ML (2004). Simulation of potential range expansion of oak disease caused by *Phytophthora cinnamomi* under climate change. Glob Chang Biol.

[CR9] Böhm J, Hahn A, Schubert R, Bahnweg G, Adler N, Nechwatal J, Oehlmann R, Oßwald W (1999). Real-time quantitative PCR: DNA determination in isolated spores of the mycorrhizal fungus *Glomus mosseae* and monitoring of *Phytophthora infestans* and *Phytophthora citricola* in their respective host plants. J Phytopathol.

[CR10] Boonham N, Glover R, Tomlinson J, Mumford R (2008). Exploiting generic platform technologies for the detection and identification of plant pathogens. Eur J Plant Pathol.

[CR11] Botella L, Bačová A, Dvořák M, Kudláček T, Pepori AL, Santini A, Ghelardini L, Luchi N (2019). Detection and quantification of the air inoculum of *Caliciopsis pinea* in a plantation of *Pinus radiata* in Italy. iForest.

[CR12] Boutigny AL, Guinet C, Vialle A, Hamelin RC, Andrieux A, Frey P, Husson C, Ioos R (2013). Optimization of a real-time PCR assay for the detection of the quarantine pathogen *Melampsora medusae* f. sp. *deltoidae*. Fungal Biol.

[CR13] Boutigny AL, Guinet C, Vialle A, Hamelin R, Frey P, Ioos R (2013). A sensitive real-time PCR assay for the detection of the two *Melampsora medusae* formae speciales on infected poplar leaves. Eur J Plant Pathol.

[CR14] Brodde L, Adamson K, Camarero JJ, Castaño C, Drenkhan R, Lehtijarvi A, Luchi N, Migliorini D, Sánchez-Miranda A, Stenlid J, Özdağ S, Oliva J (2019). Diplodia tip blight on its way to the north: drivers of disease emergence in northern Europe. Front Plant Sci.

[CR15] Bulman SR, McDougal RL, Hill K, Lear G (2018). Opportunities and limitations for DNA metabarcoding in Australasian plant-pathogen biosecurity. Australas Plant Path.

[CR16] Burgess T, Wingfield MJ (2002). Quarantine is important in restricting the spread of exotic seed-borne tree pathogens in the southern hemisphere. Int For Rev.

[CR17] Burgess TI, Crous CJ, Slippers B, Hantula J, Wingfield MJ (2016). Tree invasions and biosecurity: eco-evolutionary dynamics of hitchhiking fungi. AoB Plants.

[CR18] Bustin S, Huggett J (2017). qPCR primer design revisited. Biomol Detect Quantif.

[CR19] Bustin SA, Beaulieu JF, Huggett J, Jaggi R, Kibenge FS, Olsvik PA, Penning LC, Toegel S (2010). MIQE précis: practical implementation of minimum standard guidelines for fluorescence-based quantitative real-time PCR experiments. BMC Mol Biol.

[CR20] Catal M, Adams GC, Chastagner GA (2001) Detection, identification and quantification of latent needlecast pathogens and endophytes in symptomless conifer foliage by PCR and Dot-blot assays. In: Forest research Institute Res. Papers. Proceedings of the IUFRO Working Party 7.02.02 Shoot and foliage Diseases, 2001. Hyytiälä, Finland, 164–168

[CR21] Català S, Berbegal M, Pérez-Sierra A, Abad-Campos P (2016). Metabarcoding and development of new real-time specific assays reveal Phytophthora species diversity in holm oak forests in eastern Spain. Plant Pathol.

[CR22] Chandelier A, André F, Laurent F (2010). Detection of *Chalara fraxinea* in common ash (*Fraxinus excelsior*) using real time PCR. Forest Pathol.

[CR23] Chandelier A, Helson M, Dvořák M, Gischer F (2014). Detection and quantification of airborne inoculum of *Hymenoscyphus pseudoalbidus* using real-time PCR assays. Plant Pathol.

[CR24] Chapman D, Purse BV, Roy HE, Bullock JM (2017). Global trade networks determine the distribution of invasive non-native species. Glob Ecol Biogeogr.

[CR25] Chen W, Hambleton S, Seifert KA, Carisse O, Diarra MS, Peters RD, Lowe C, Chapados JT, Lévesque CA (2018). Assessing performance of spore samplers in monitoring aeromycobiota and fungal plant pathogen diversity in Canada. Appl Environ Microbiol.

[CR26] Coutinho TA, Steenkamp ET, Mongwaketsi K, Wilmot M, Wingfield MJ (2007). First outbreak of pitch canker in a south African pine plantation. Australas Plant Pathol.

[CR27] D’Amico L, Motta E, Annesi T, Scire’ M, Luchi N, Hantula J, Korhonen K, Capretti P (2007). The North American P group of *Heterobasidion annosum* s.l. is widely distributed in *Pinus pinea* forests of the western coast of Central Italy. For Pathol.

[CR28] DeScenzo RA, Harrington TC (1994). Use of (CAT)_5_ as a DNA fingerprinting probe for fungi. Phytopathology.

[CR29] Desprez-Loustau ML, Marçais B, Nageleisen LM, Piou D, Vannini A (2006). Interactive effects of drought and pathogens in forest trees. Ann For Sci.

[CR30] EPPO (2014). PM 7/98 (2) specific requirements for laboratories preparing accreditation for a plant pest diagnostic activity. EPPO Bull.

[CR31] Eschen R, Britton K, Brockerhoff E, Burgess T, Dalley V, Epanchin-Niell RS, Gupta K, Hardy G, Huang Y, Kenis M, Kimani E, Li HM, Olsen S, Ormrod R, Otieno W, Sadof C, Tadeu E, Theyse M (2015). International variation in phytosanitary legislation and regulations governing importation of plants for planting. Environ Sci Pol.

[CR32] Eschen R, Douma JC, Grégoire J-C, Mayer F, Rigaux L, Potting RPJ (2017). A risk categorisation and analysis of the geographic and temporal dynamics of the European import of plants for planting. Biol Invasions.

[CR33] Eschen R, O’Hanlon R, Santini A, Vannini A, Roques A, Kirichenko N, Kenis M (2019). Safeguarding global plant health: the rise of sentinels. J Pest Sci.

[CR34] Fabre B, Piou D, Desprez-Loustau ML, Marçais B (2011). Can the emergence of pine *Diplodia* shoot blight in France be explained by changes in pathogen pressure linked to climate change?. Glob Chang Biol.

[CR35] Feau N, Decourcelle T, Husson C, Desprez-Loustau M-L, Dutech C (2011). Finding single copy genes out of sequenced genomes for multilocus phylogenetics in non-model fungi. PLoS One.

[CR36] Fisher MC, Henk DA, Briggs CJ, Brownstein JS, Madoff LC, McCraw SL, Gurr SJ (2012). Emerging fungal threats to animal, plant and ecosystem health. Nature.

[CR37] Fourrier C, Antoine S, Piou D, Ioos R (2015). Rapid detection of *Fusarium circinatum* propagules on trapped pine beetles. Forest Pathol.

[CR38] Franić I, Prospero S, Hartmann M, Allan E, Auger-Rozenberg M-A, Grünwald NJ, Kenis M, Roques A, Schneider S, Sniezko R, Williams W, Eschen R (2019) Are traded forest tree seeds a potential source of nonnative pests? Ecol Appl:e01971. 10.1002/eap.1971 10.1002/eap.1971 31302945

[CR39] Gachon C, Mingam A, Charrier B (2004). Real-time PCR: what relevance to plant studies?. J Exp Bot.

[CR40] Ghelardini L, Pepori AL, Luchi N, Capretti P, Santini A (2016). Drivers of emerging fungal diseases of forest trees. For Ecol Manag.

[CR41] Goggioli V, Capretti P, Hamelin RC, Vendramin GG (1998). Isozyme and RAPD polymorphisms in *Heterobasidion annosum* in Italy. Eur J For Pathol.

[CR42] Grosdidier M, Aguayo J, Marçais B, Ioos R (2017). Detection of plant pathogens using real-time PCR: how reliable are late Ct values?. Plant Pathol.

[CR43] Grosdidier M, Ioos R, Husson C, Cael O, Scordia T, Marçais B (2018). Tracking the invasion: dispersal of *Hymenoscyphus fraxineus* airborne inoculum at different scales. FEMS Microbiol Ecol.

[CR44] Halk EL, De Boer SH (1985). Monoclonal antibodies in plant disease research. Annu Rev Phytopathol.

[CR45] Halmschlager E, Messner R, Kowalski T, Prillinger H (1995). Differentiation of *Ophiostoma piceae* and *Ophiostoma quercus* by morphology and RAPD analysis. Syst Appl Microbiol.

[CR46] Hansson P, Wang XR, Szmidt AE, Karlman M (1996). RAPD variation in *Gremmeniella abietina* attacking *Pinus sylvestris* and *Pinus contorta* in northern Sweden. Eur J For Pathol.

[CR47] Hantula J, Dusabenyagasani M, Hamelin RC (1996). Random amplified microsatellites (RAMS) - a novel method for characterizing genetic variation within fungi. Eur J For Pathol.

[CR48] Harrison C, Tomlinson J, Ostoja-Starzewska S, Boonham N (2017). Evaluation and validation of a loop-mediated isothermal amplification test kit for detection of *Hymenoscyphus fraxineus*. Eur J Plant Pathol.

[CR49] Hayden KJ, Rizzo D, Tse J, Garbelotto M (2004). Detection and quantification of *Phytophthora ramorum* from California forests using a real-time polymerase chain reaction assay. Phytopathology.

[CR50] Heller WP, Keith LM (2018). Real-time PCR assays to detect and distinguish the rapid ʻōhiʻa death pathogens *Ceratocystis lukuohia* and *C. huliohia*. Phytopathology.

[CR51] Hellman JJ, Byers JE, Bierwagen B (2008). Five potential consequences of climate change for invasive species. Conserv Biol.

[CR52] Hintz WE, Jeng RS, Hubbes M, Horgen PA (1991). Identification of three populations of *Ophiostoma ulmi* (aggressive subgroup) by mitochondrial DNA restriction-site mapping and nuclear DNA-fingerprinting. Exp Mycol.

[CR53] Hughes KJD, Giltrap PM, Barton VC, Hobden E, Tomlinson JA, Barber P (2006). On-site real-time PCR detection of *Phytophthora ramorum* causing dieback of *Parrotia persica* in the UK. Plant Pathol.

[CR54] Husson C, Ioos R, Andrieux A, Frey P (2013). Development and use of new sensitive molecular tools for diagnosis and detection of Melampsora rusts on cultivated poplar. For Pathol.

[CR55] Husson C, Aguayo J, Revellin C, Frey P, Ioos R, Marçais B (2015). Evidence for homoploid speciation in *Phytophthora alni* supports taxonomic reclassification in this species complex. Fungal Genet Biol.

[CR56] Ioos R, Husson C, Andrieux A, Frey P (2005). SCAR-based PCR primers to detect the hybrid pathogen *Phytophthora alni* and its subspecies causing alder disease in Europe. Eur J Plant Pathol.

[CR57] Ioos R, Laugustin L, Schenck N, Rose S, Husson C, Frey P (2006). Usefulness of single copy genes containing introns in Phytophthora for the development of detection tools for the regulated species *P. ramorum* and *P. fragariae*. Eur J Plant Pathol.

[CR58] Ioos R, Fourrier C, Iancu G, Gordon TR (2009). Sensitive detection of *Fusarium circinatum* in pine seed by combining an enrichment procedure with a real-time polymerase chain reaction using dual-labeled probe chemistry. Phytopathology.

[CR59] Ioos R, Kowalski T, Husson C, Holdenrieder O (2009). Rapid in planta detection of *Chalara fraxinea* by a real-time PCR assay using a dual-labelled probe. Eur J Plant Pathol.

[CR60] Ioos R, Fabre B, Saurat C, Fourrier C, Frey P, Marçais B (2010). Development, comparison, and validation of real-time and conventional PCR tools for the detection of the fungal pathogens causing brown spot and red band needle blights of pine. Phytopathology.

[CR61] Ioos R, Aloi F, Piškur B, Guinet C, Mullett M, Berbegal M, Bragança H, Cacciola SO, Oskay F, Cornejo C, Adamson K, Douanla-Meli C, Kačergius A, Martínez-Álvarez P, Nowakowska JA, Luchi N, Vettraino AM, Ahumada R, Pasquali M, Fourie G, Kanetis L, Alves A, Ghelardini L, Dvořák M, Sanz-Ros A, Diez JJ, Baskarathevan J, Aguayo J (2019). Are PCR-based diagnostic protocols easily transferable? An international collaborative case study assessing protocols targeting the quarantine pine pathogen *Fusarium circinatum*. Sci Rep.

[CR62] Ioos R, Chrétien P, Perrault J, Jeandel C, Dutech C, Gonthier P, Sillo F, Hietala AM, Solheim H, Hubert J (2019). Multiplex real-time PCR assays for the detection and identification of Heterobasidion species attacking conifers in Europe. Plant Pathol.

[CR63] Jeng RS, Duchesne LC, Sabourln M, Hubbes M (1991). Mitochondrial DNA restriction fragment length polymorphisms of aggressive and non-aggressive isolates of *Ophiostoma ulmi*. Mycol Res.

[CR64] Karlsson JO (1994). Genetic variation in *Heterobasidion annosum* detected with M13 fingerprinting and ribosomal RNA probes. Exp Mycol.

[CR65] Karlsson JO, Stenlid J (1991). Pectic isozyme profiles of intersterility groups in *Heterobasidion annosum*. Mycol Res.

[CR66] Kirisits T, Lieutier F, Day KR, Battisti A, Gŕegoire J-C, Evans HF (2004). Fungal associates of European bark beetles with special emphasis on the Ophiostomatoid fungi. Bark and wood boring insects in living trees in Europe, a synthesis.

[CR67] Klapwijk MJ, Hopkins AJ, Eriksson L, Pettersson M, Schroeder M, Lindelöw Å, Rönnberg J, Keskitalo EC, Kenis M (2016). Reducing the risk of invasive forest pests and pathogens: combining legislation, targeted management and public awareness. Ambio.

[CR68] Lamarche J, Potvin A, Pelletier G, Stewart D, Feau N, Alayon DI, Dale AL, Coelho A, Uzunovic A, Bilodeau GJ, Brière SC, Hamelin RC, Tanguay P (2015). Molecular detection of 10 of the most unwanted alien forest pathogens in Canada using real-time PCR. PLoS One.

[CR69] Liebhold AM, Brockerhoff EG, Garrett LJ, Parke JL, Britton KO (2012). Live plant imports: the major pathway for forest insect and pathogen invasions of the US. Front Ecol Environ.

[CR70] Livak KJ, Flood SJA, Marmaro J, Giusti W, Deetz K (1995). Oligonucleotides with fluorescent dyes at opposite ends provide a quenched probe system useful for detecting PCR product and nucleic acid hybridization. PCR Methods Appl.

[CR71] Luchi N, Capretti P, Pinzani P, Orlando C, Pazzagli M (2005). Real-time PCR detection of *Biscogniauxia mediterranea* in symptomless oak tissue. Lett Appl Microbiol.

[CR72] Luchi N, Capretti P, Surico G, Orlando C, Pazzagli M, Pinzani P (2005). A real-time quantitative PCR assay for the detection of *Sphaeropsis sapinea* from inoculated *Pinus nigra* shoots. J Phytopathol.

[CR73] Luchi N, Capretti P, Vettraino AM, Vannini A, Pinzani P, Pazzagli M (2006). Early detection of *Biscogniauxia nummularia* in symptomless European beech (*Fagus sylvatica* L.) by TaqMan™ real-time PCR. Lett Appl Microbiol.

[CR74] Luchi N, Pratesi N, Simi L, Pazzagli M, Capretti P, Scala A, Slippers B, Pinzani P (2011). High-resolution melting analysis: a new molecular approach for the early detection of *Diplodia pinea* in Austrian pine. Fungal Biol.

[CR75] Luchi N, Mancini V, Feducci M, Santini A, Capretti P (2012). *Leptoglossus occidentalis* and *Diplodia pinea*: a new insect-fungus association in Mediterranean forests. Forest Pathol.

[CR76] Luchi N, Ghelardini L, Belbahri L, Quartier M, Santini A (2013). Rapid detection of *Ceratocystis platani* inoculum by quantitative real-time PCR. Appl Environ Microbiol.

[CR77] Luchi N, Capretti P, Pazzagli M, Pinzani P (2016). Powerful qPCR assays for the early detection of latent invaders: interdisciplinary approaches in clinical cancer research and plant pathology. Appl Microbiol Biotechnol.

[CR78] Luchi N, Pepori AL, Bartolini P, Ioos R, Santini A (2018). Duplex real-time PCR assay for the simultaneous detection of *Caliciopsis pinea* and *Fusarium circinatum* in pine samples. Appl Microbiol Biotechnol.

[CR79] Luchi N, Santini A, Salvianti F, Pinzani P, Biassoni R, Raso A (2020). Early detection of fungal plant pathogens by real-time quantitative PCR: the case of *Diplodia sapinea* on pine. Quantitative real-time PCR. Methods in molecular biology.

[CR80] Maresi G, Luchi N, Pinzani P, Pazzagli M, Capretti P (2007). Detection of *Diplodia pinea* in asymptomatic pine shoots and its relation to the normalized insolation index. Forest Pathol.

[CR81] Marthey S, Aguileta G, Rodolphe F, Gendrault A, Giraud T, Fournier E, Lopez-Villavicencio M, Gautier A, Lebrun M-H, Chiapello H (2008). FUNYBASE: a FUNgal phYlogenomic dataBASE. BMC Bioinformatics.

[CR82] Migliorini D, Ghelardini L, Tondini E, Luchi N, Santini A (2015). The potential of symptomless potted plants for carrying invasive soilborne plant pathogens. Divers Distrib.

[CR83] Migliorini D, Ghelardini L, Luchi N, Capretti P, Onorati M, Santini A (2019). Temporal evolution of airborne *Phytophthora* spp. in a woody plant nursery area using real time-PCR. Aerobiologia.

[CR84] Miller SA, Martin RR (1998). Molecular diagnosis of plant diseases. Annu Rev Phytopathol.

[CR85] Möller M, Stukenbrock EH (2017). Evolution and genome architecture in fungal plant pathogens. Nat Rev Microbiol.

[CR86] Mullett MS, Adamson K, Bragança H, Bulgakov TS, Georgieva M, Henriques J, Jürisoo L, Laas M, Drenkhan R (2018). New country and regional records of the pine needle blight pathogens *Lecanosticta acicola*, *Dothistroma septosporum* and *Dothistroma pini*. Forest Pathol.

[CR87] Notomi T, Okayama H, Masubuchi H, Yonekawa T, Watanabe K, Amino N, Hase T (2000). Loop-mediated isothermal amplification of DNA. Nucleic Acids Res.

[CR88] Oak SW, Elledge AH, Yockey EK, Smith WD, Tkacz BM (2008) *Phytophthora ramorum* early detection surveys for forests in the United States, 2003–2006. In: Frankel SJ, Kliejunas JT, Palmieri K M. Proceedings of the sudden oak death third science symposium. Gen. Tech. Rep. PSW-GTR-214. Albany, CA: U.S. Department of Agriculture, Forest Service, Pacific Southwest Research Station. pp. 413–416

[CR89] Old KM, Moran GF, Bell JC (1984). Isozyme variability among isolates of *Phytophthora cinnamomi* from Australia and Papua New Guinea. Can J Bot.

[CR90] Olson A, Aerts A, Asiegbu F, Belbahri L, Bouzid O, Broberg A, Canbäck B, Coutinho PM, Cullen D, Dalman K, Deflorio G, van Diepen LT, Dunand C, Duplessis S, Durling M, Gonthier P, Grimwood J, Fossdal CG, Hansson D, Henrissat B, Hietala A, Himmelstrand K, Hoffmeister D, Högberg N, James TY, Karlsson M, Kohler A, Kües U, Lee YH, Lin YC, Lind M, Lindquist E, Lombard V, Lucas S, Lundén K, Morin E, Murat C, Park J, Raffaello T, Rouzé P, Salamov A, Schmutz J, Solheim H, Ståhlberg J, Vélëz H, de Vries RP, Wiebenga A, Woodward S, Yakovlev I, Garbelotto M, Martin F, Grigoriev IV, Stenlid J (2012). Insight into trade-off between wood decay and parasitism from the genome of a fungal forest pathogen. New Phytol.

[CR91] Otrosina WJ, Ghase TE, Gobb FW, Korhonen K (1992). Allozyme differentiation of intersterility groups of *Heterobasidion annosum* isolated from conifers in the Western United States. Phytopathol.

[CR92] Palm M, Rossman AY, Ruiz GM, Carlton JT (2003). Invasion pathways of terrestrial plant-inhabiting fungi. Invasive species: vectors and management strategies.

[CR93] Pipe ND, Buck KW, Brasier CM (1995). Molecular relationships between *Ophiostoma ulmi* and the NAN and EAN races of *O. novo-ulmi* determined by RAPD markers. Mycol Res.

[CR94] Raddi S, Santini A, Casini N (1994) Comparison of enzymatic polymorphism in different Seiridium isolates. Proc Congr Medit Phytopathol Union, 9th, Kusadasi, pp 281–285. Izmir: Turk Phytopathol Soc

[CR95] Santini A, Capretti P (2000). Analysis of the Italian population of *Ceratocystis fimbriata* f. sp. *platani* using RAPD and minisatellite markers. Plant Pathol.

[CR96] Santini A, Ghelardini L (2015). Plant pathogen evolution and climate change. CAB Reviews.

[CR97] Santini A, Montaghi A, Vendramin GG, Capretti P (2005). Analysis of the Italian Dutch elm disease fungal population. J Phytopathol.

[CR98] Santini A, Ghelardini L, De Pace C, Desprez-Loustau ML, Capretti P, Chandelier A, Cech T, Chira D, Diamandis S, Gaitniekis T, Hantula J, Holdenrieder O, Jankovsky L, Jung T, Jurc D, Kirisits T, Kunca A, Lygis V, Malecka M, Marçais B, Schmitz S, Schumacher J, Solheim H, Solla A, Szabò I, Tsopelas P, Vannini A, Vettraino AM, Webber J, Woodward S, Stenlid J (2013). Biogeographical patterns and determinants of invasion by forest pathogens in Europe. New Phytol.

[CR99] Santini A, Liebhold A, Migliorini D, Woodward S (2018). Tracing the role of human civilization in the globalization of plant pathogens. ISME J.

[CR100] Schenck N, Fourrier-Jeandel C, Ioos R (2016). A robust and specific real-time PCR tool for the detection of *Phytophthora lateralis* in plant tissues. Eur J Plant Pathol.

[CR101] Schmitt I, Crespo A, Divakar PK, Fankhauser JD, Herman-Sackett E, Kalb K, Nelsen MP, Nelson NA, Rivas-Plata E, Shimp AD, Widhelm T, Lumbsch HT (2009). New primers for promising single-copy genes in fungal phylogenetics and systematics. Persoonia.

[CR102] Schneider S, Jung E, Queloz V, Meyer JB, Rigling D (2019). Detection of pine needle diseases caused by *Dothistroma septosporum*, *Dothistroma pini* and *Lecanosticta acicola* using different methodologies. Forest Pathol.

[CR103] Schoch CL, Seifert KA, Huhndorf S, Robert V, Spouge JL, Levesque CA, Chen W, Consortium F B (2012). Nuclear ribosomal internal transcribed spacer (ITS) region as a universal DNA barcode marker for fungi. Proc Natl Acad Sci U S A.

[CR104] Schweigkofler W, O’Donnell K, Garbelotto M (2004). Detection and quantification of airborne conidia of *Fusarium circinatum*, the causal agent of pine pitch canker, from two California sites by using a real-time PCR approach combined with a simple spore trapping method. Appl Environ Microbiol.

[CR105] Sicard A, Zeilinger AR, Vanhove M, Schartel TE, Beal DJ, Daugherty MP, Almeida RPP (2018). *Xylella fastidiosa*: insights into an emerging plant pathogen. Annu Rev Phytopathol.

[CR106] Sillo F, Giordano L, Gonthier P (2018). Fast and specific detection of the invasive forest pathogen *Heterobasidion irregulare* through a loop-mediated isothermal AMPlification (LAMP) assay. For Pathol.

[CR107] Slippers B, Wingfield MJ (2007). Botryosphaeriaceae as endophytes and latent pathogens of woody plants: diversity, ecology and impact. Fungal Biol Rev.

[CR108] Slippers B, Boissin E, Phillips AJ, Groenewald JZ, Lombard L, Wingfield MJ, Postma A, Burgess T, Crous PW (2013). Phylogenetic lineages in the Botryosphaeriales: a systematic and evolutionary framework. Stud Mycol.

[CR109] Slippers B, Crous PW, Jami F, Groenewald JZ, Wingfield MJ (2017). Diversity in the Botryosphaeriales: looking back, looking forward. Fungal Biol.

[CR110] Smith H, Wingfield MJ, Crous PW, Coutinho TA (1996). *Sphaeropsis sapinea* and *Botryosphaeria dothidea* endophytic in *Pinus* spp. and *Eucalyptus* spp. in South Africa. S Afr J Bot.

[CR111] Stanosz GR, Blodgett JT, Smith DR, Kruger EL (2001). Water stress and *Sphaeropsis sapinea* as a latent pathogen of red pine seedlings. New Phythol.

[CR112] Stenlid J, Karlsson JO, Högberg N (1994). Intraspecific genetic variation in *Heterobasidion annosum* revealed by amplification of mini satellite DNA. Mycol Res.

[CR113] Storer AJ, Gordon TR, Clark SL (1998). Association of the pitch canker fungus*, Fusarium subglutinans* f. sp. *pini*, with Monterey pine seeds and seedlings in California. Plant Pathol.

[CR114] Swett CL, Gordon TR (2012). First report of grass species (Poaceae) as naturally occurring hosts of the pine pathogen *Gibberella circinata*. Plant Dis.

[CR115] Swett CL, Porter B, Fourie G, Steenkamp ET, Gordon TR, Wingfield MJ (2014). Association of the pitch canker pathogen *Fusarium circinatum* with grass hosts in commercial pine production areas of South Africa. South forests.

[CR116] Thompson RN, Gilligan CA, Cunniffe NJ (2016). Detecting presymptomatic infection is necessary to forecast major epidemics in the earliest stages of infectious disease outbreaks. PLoS Comput Biol.

[CR117] Tomlinson JA, Boonham N, Hughes KJD, Griffin RL, Barker I (2005). On-site DNA extraction and real-time PCR for detection of *Phytophthora ramorum* in the field. Appl Environ Microbiol.

[CR118] Tomlinson JA, Dickinson M, Hobden E, Robinson S, Giltrap PM, Boonham N (2010). A five-minute DNA extraction method for expedited detection of *Phytophthora ramorum* following prescreening using *Phytophthora* spp. lateral flow devices. J Microbiol Methods.

[CR119] Tooley PW, Martin FN, Carras MM, Frederick RD (2006). Real-time fluorescent polymerase chain reaction detection of *Phytophthora ramorum* and *Phytophthora pseudosyringae* using mitochondrial gene regions. Phytopathology.

[CR120] Torrance L (1995). Use of monoclonal antibodies in plant pathology. Eur J Plant Pathol.

[CR121] Tsao PH (1970). Selective media for isolation of pathogenic fungi. Annu Rev Phytopathol.

[CR122] Vander Heyden Y, Nijhuis A, Smeyers-Verbeke J, Vandeginste BG, Massart DL (2001). Guidance for robustness/ruggedness tests in method validation. J Pharm Biomed Anal.

[CR123] Vasiliauskas R, Stenlid J (1997). Population structure and genetic variation in *Nectria fuckeliana*. Can J Bot.

[CR124] Vettraino A, Roques A, Yart A, Fan J-T, Sun J-H, Vannini A (2015). Sentinel trees as a tool to forecast invasions of alien plant pathogens. PLoS One.

[CR125] Vettraino AM, Li H-M, Eschen R, Morales-Rodriguez C, Vannini A (2017). The sentinel tree nursery as an early warning system for pathway risk assessment: fungal pathogens associated with Chinese woody plants commonly shipped to Europe. PLoS One.

[CR126] Vialle A, Feau N, Frey P, Bernier L, Hamelin RC (2013). Phylogenetic species recognition reveals host-specific lineages among poplar rust fungi. Mol Phylogenet Evol.

[CR127] Vicent A, Blasco J (2017). When prevention fails. Towards more efficient strategies for plant disease eradication. New Phytol.

[CR128] Vicente C, Espada M, Vieira P, Mota M (2012). Pine wilt disease: a threat to European forestry. Eur J Plant Pathol.

[CR129] Villari C, Tomlinson JA, Battisti A, Boonham N, Capretti P, Faccoli M (2013). Use of loop-mediated isothermal amplification for detection of *Ophiostoma clavatum*, the primary blue stain fungus associated with *Ips acuminatus*. Appl Environ Microbiol.

[CR130] Vincelli P, Tisserat N (2008). Nucleic acid–based pathogen detection in applied plant pathology. Plant Dis.

[CR131] White TJ, Bruns TD, Lee SB, Taylor JW, Innis MA, Gelfand DH, Sninsky JJ, White TJ (1990). Amplification and direct sequencing of fungal ribosomal RNA genes for phylogenetics. PCR protocols: a guide to methods and applications.

[CR132] Wingfield MJ, De Beer ZW, Slippers B, Wingfield BD, Groenewald JZ, Lombard L, Crous PW (2011). One fungus, one name promotes progressive plant pathology. Mol Plant Pathol.

[CR133] Winton LM, Stone JK, Watrud LS, Hansen EM (2002). Simultaneous one-tube quantification of host and pathogen DNA with real-time polymerase chain reaction. Phytopathol.

[CR134] Wittenberg R, Cock MJW (2001). Invasive alien species: a toolkit of best prevention and management practices.

[CR135] Witthuhn RC, Wingfield BD, Wingfield MJ, Harrington TC (1999). PCR-based identification and phylogeny of species of *Ceratocystis* sensu stricto. Mycol Res.

[CR136] Zhou S, Stanosz GR (2001). Primers for amplification of mt SSU rDNA, and a phylogenetic study of Botryosphaeria and associated anamorphic fungi. Mycol Res.

